# Implications of survey effort on estimating demographic parameters of a long‐lived marine top predator

**DOI:** 10.1002/ece3.4512

**Published:** 2018-10-03

**Authors:** John Symons, Kate R. Sprogis, Lars Bejder

**Affiliations:** ^1^ Cetacean Research Unit School of Veterinary and Life Sciences Murdoch University Murdoch Western Australia Australia; ^2^ Zoophysiology Department of Bioscience Aarhus University Aarhus Denmark; ^3^ Marine Mammal Research Program Hawaii Institute of Marine Biology University of Hawaii at Manoa Honolulu Hawaii

**Keywords:** bottlenose dolphin, conservation, home range, marine mammal, population parameters, reproductive biology, survival, wildlife management, abundance

## Abstract

Effective management of wildlife populations rely on knowledge of their abundance, survival, and reproductive rates. Maintaining long‐term studies capable of estimating demographic parameters for long‐lived, slow‐reproducing species is challenging. Insights into the effects of research intensity on the statistical power to estimate demographic parameters are limited. Here, we investigate implications of survey effort on estimating abundance, home range sizes, and reproductive output of Indo‐Pacific bottlenose dolphins (*Tursiops aduncus*), using a 3‐year subsample of a long‐term, capture–recapture study off Bunbury, Western Australia. Photo‐identification on individual dolphins was collected following Pollock's Robust Design, where seasons were defined as “primary periods”, each consisting of multiple “secondary periods.” The full dataset consisted of 12 primary periods and 72 secondary periods, resulting in the study area being surveyed 24 times/year. We simulated reduced survey effort by randomly removing one, two, or three secondary periods per primary period. Capture–recapture models were used to assess the effect of survey intensity on the power to detect trends in population abundance, while individual dolphin sighting histories were used to assess the ability to conduct home range analyses. We used sighting records of adult females and their calving histories to assess survey effort on quantifying reproductive output. A 50% reduction in survey effort resulted in (a) up to a 36% decline in population abundance at the time of detection; (b) a reduced ability to estimate home range sizes, by increasing the time for individuals to be sighted on ≥30 occasions (an often‐used metric for home range analyses) from 7.74 to 14.32 years; and (c) 33%, 24%, and 33% of annual calving events across three years going undocumented, respectively. Results clearly illustrate the importance of survey effort on the ability to assess demographic parameters with clear implications for population viability analyses, population forecasting, and conservation efforts to manage human–wildlife interactions.

## 1 INTRODUCTION

The conservation and management of wildlife populations of commercial, natural, and cultural significance is a challenging and complex issue (McShane et al., 2011; Pressey, Cabeza, Watts, Cowling, & Wilson, 2007). Many populations face increasing pressures from natural and human sources, for example, overexploitation (Burgess, Polasky, & Tilman, 2013; Worm et al., 2013), climate change (Mawdsley, O'Malley, & Ojima, 2009), habitat degradation (Brooks et al., 2002; Hoekstra, Boucher, Ricketts, & Roberts, 2005), by‐catch (Hall, Alverson, & Metuzals, 2000; Read, Drinker, & Northridge, 2006), and unsustainable tourism practices (Van der Duim & Caalders, 2002), thus increasing the need for conservation efforts (Kraus et al., 2005; Sadovy de Mitcheson et al., 2013; Spangenberg, 2007; Wallace & Saba, 2009). Wildlife managers rely on accurate information on population demographic parameters (e.g., abundance, survival rate, and reproductive rate) to effectively implement informed management decisions that aim to optimize viable populations (Akçakaya, 2000; Baum et al., 2003; Crouse, Crowder, & Caswell, 1987).

Long‐term monitoring programs serve a critical role in understanding ecological systems and for the development of informed wildlife policymaking (Bejder et al., 2006; Editorial 2017; Hughes et al., 2017; Mann & Karniski, 2017; Mullon, Freon, & Cury, 2005; Wittemyer, Daballen, & Douglas‐Hamilton, 2013). Unfortunately, long‐term studies of long‐lived species are both resource and time‐demanding (Tyne et al., 2016), and securing their ongoing funding is often a challenge (Editorial 2017; Hughes et al., 2017; Williams & Thomas, 2009). To enhance both the cost‐effectiveness and the biological inferences of long‐term studies, they often aim to collect data streams that are useful for assessments of multiple demographic parameters (Christiansen, Bertulli, Rasmussen, & Lusseau, 2015; Fujiwara & Caswell, 2001; Moss, 2001; Wierucka, Halupka, Klimczuk, & Sztwiertnia, 2016; Wittemyer et al., 2013). However, the importance of a specific demographic parameter to the long‐term viability of a population is highly variable (Heppell, Caswell, & Crowder, 2000; Oli & Dobson, 2003).

Demographic parameters vary between populations of the same species and between species (see McMahon, Burton, & Bester, 2003; Moss, 2001; Nicholson, Bejder, Allen, Krützen, & Pollock, 2012; Sprogis et al., 2016a; Wittemyer et al., 2013). Inferring demographic parameters estimated for one population to another population of a similar species may result in inaccurate conclusions when evaluating their long‐term viability (Manlik et al., 2016). The time required to accurately assess demographic parameters of long‐lived, slowly reproducing species can take years (Mann, Connor, Barre, & Heithaus, 2000; Moss, 2001). Optimizing the power of a survey design to meet its research objectives is therefore critical (Hawkins et al., 2017). While several monitoring studies have quantified the power of survey designs to detect trends in population abundance (e.g., Ansmann, Lanyon, Seddon, & Parra, 2013; Brown, Bejder, Pollock, & Allen, 2015; Parra, Corkeron, & Marsh, 2006; Tyne et al., 2016; Wilson, Hammond, & Thompson, 1999), they have ignored the potential implications of survey design for the assessment of additional demographic parameters such as reproductive and survival rates.

The waters off Bunbury, Western Australia, are home to a resident population of Indo‐Pacific bottlenose dolphins (*Tursiops aduncus*). Abundance estimates vary seasonally from a minimum of 76 dolphins (95% CI 68 to 85) in the winter to a maximum of 185 dolphins (95% CI 171 to 199) in the summer (Sprogis et al., 2016a). Here, individuals utilize the area differently, with animals sighted in open waters having larger home ranges than those utilizing predominately inshore waterways (Sprogis, Smith, Rankin, MacLeod, & Bejder, 2016b). The Port of Bunbury is currently the fourth largest port in Western Australia (Ports Australia 2013) and is expanding its capacity to a greater extent to support growing recreational and commercial vessel operations (Australian Government Department of the Environment 2016; Taylor Burrell Barnett 2015). In addition, dolphin‐based tourism (including boat‐based dolphin eco‐cruises, swim‐with‐dolphin tours, and licensed food provisioning of wild dolphins) represents a substantial proportion of Bunbury's tourism economy (Smith, 2012). Consequently, the Bunbury dolphin population is exposed to multiple sources of human activities, including recreational vessel traffic (Jensen et al., 2009), commercial shipping traffic, commercial tourism, and both licensed (legal) and unlicensed (illegal) food provisioning (Arcangeli & Crosti, 2009; Smith, 2012).

The long‐term viability of the Bunbury dolphin population is projected to decline by 50% in the next 20 years (Manlik et al., 2016). Low reproductive rates have been identified as the leading cause for the decline (Manlik et al., 2016). Historically, the Bunbury dolphin population has served as a “source” population for the larger meta‐population along the southwestern Australian coast (Manlik et al., [Link]In Press), and more recently, the abundance and temporary emigration of the population are also influenced by climate variability (Sprogis, Christiansen, Wandres, & Bejder, 2018), thus raising additional conservation concerns for the overall meta‐population viability. Therefore, it is important to understand both the ability to detect trends in population abundance and to quantify demographic parameters for this population to best inform management.

Here, we utilized a 3‐year subsample of an ongoing long‐term capture–recapture photo‐identification study focussing on the local dolphin population (Smith, Pollock, Waples, Bradley, & Bejder, 2013; Sprogis et al., 2016a, 2018). This study conformed to the structure of Pollock's Robust Design (hereafter referred to as “Robust Design”; Pollock, 1982). The objectives were to assess how various levels of survey effort impacted our ability to (a) detect trends in population abundance; (b) quantify apparent survival rates; (c) conduct home range size analyses; (d) detect calving events; and (e) quantify the uncertainty surrounding a calf's period of birth. This study provides novel insights into the implications of survey effort to estimate several key demographic parameters of a long‐lived species.

## 2 METHODS

### 2.1 Data collection

**Figure 1:**
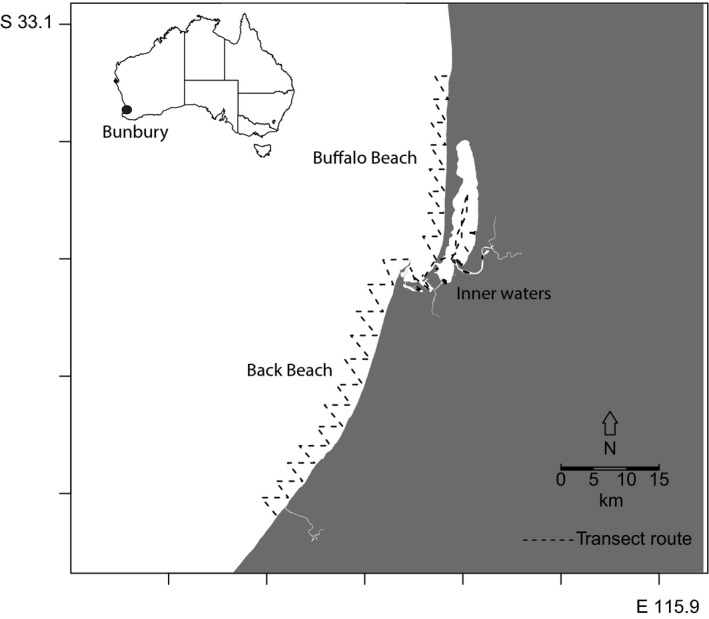
The 120‐km^2^ study area off Bunbury, Western Australia. The study area was divided into three transects (dashed lines) along which boat‐based photo‐identification capture–recapture surveys were conducted for Indo‐Pacific bottlenose dolphins: Buffalo Beach, Back Beach, and Inner waters transects

Boat‐based photo‐identification surveys for dolphins have been conducted year‐round since March 2007 with survey effort throughout the study area off Bunbury, southwestern Australia (115°63′E, 33°32′S; Figure 1). In this study, we use data from a 3‐year subsample (December 2009 to November 2012) of the long‐term dolphin monitoring program. The study area encompassed a region of 120 km^2^, extending 2 km from shore and covering 50 km along the coast. Surveys were conducted using a 5 m research boat powered with a four‐strike 80 HP engine and traveling at 8–12 knots following predetermined transect routes. Three systematic zig‐zag transects covered the study area (Figure 1): Buffalo Beach, Back Beach, and Inner waters. Surveys were performed under good sea conditions of Beaufort sea states ≤3. While on survey, two to five observers (median = four) visually scanned for dolphins within approximately 250 m on either side of the vessel. When a dolphin group was encountered, a “sighting” commenced. During a dolphin group sighting, photographers using digital single‐lens reflex cameras (Nikon D300s equipped with 300 or 400 mm lenses) aimed to photograph every dolphin's dorsal fin for identification (Würsig & Jefferson, 1990). A group was defined as one or more dolphins within 100 m of any other dolphin and involved in the same or similar behavioral activity (Smith et al., 2013). For further details on data collection and study design, see Smith et al. (2013) and Sprogis et al. (2016a).

### 2.2 Capture–recapture sampling design

Boat‐based surveys conformed to the Pollock's Robust Design capture–recapture method (Pollock, 1982; Sprogis et al., 2016a). The Robust Design model is constructed of a series of “primary sampling periods” (hereafter referred to as primary periods), each consisting of multiple “secondary sampling periods” (hereafter referred to as secondary periods). A population is assumed to be open between primary periods and closed within each primary period. In Bunbury, each primary period consisted of one austral season of sampling effort: summer (December–February); autumn (March–May); winter (June–August); or spring (September–November). Each season consisted of six secondary periods. The completion of the three transect zones (Buffalo Beach, Back Beach, and Inner waters) defined a secondary period (Figure 1). Sprogis et al. (2016a) defined the Robust Design model assumptions and the steps taken to minimize violations of these assumptions in this study. As such, the full data set consisted of 12 primary sampling periods equivalent to 72 secondary sampling periods over a 3‐year period, resulting in the study area being surveyed 24 times per year.

### 2.3 Data processing

In the photographic images of dolphin dorsal fins, unique nick and notch outlines were used to identify each individual (Wursig & Wursig, 1977) to a long‐term catalogue. Two or more researchers independently conducted the fin‐matching process for each individual and ensured correct identification of individuals. Images were graded following the protocols established by Rosel et al. (2011) for distinctiveness and image quality. Individual dolphin capture histories included only individuals with distinctive dorsal fins contained within high‐quality images, following Sprogis et al. (2016a).

### 2.4 Survey sampling scenarios

We explored our ability to detect trends in population abundance, estimate apparent survival rates (i.e., the total effect including true survival and emigration), conduct home range analyses, detect reproductive events and the precision of new calves ages for four scenarios of survey effort over 3 years. Specifically:

- Original Data: The original capture history for the 12 primary periods sampled, each consisting of six secondary periods (i.e., the entire study area being surveyed 24 times per year for 3 years).
- Simulation 1: A simulated reduction of survey effort to five secondary periods per primary period, by removing one secondary period per primary period.
- Simulation 2: A simulated reduction of survey effort to four secondary periods per primary period, by removing two secondary periods per primary period.
- Simulation 3: A simulated reduction of survey effort to three secondary periods per primary period, by removing three secondary periods per primary period.

Further reduction of survey effort was not explored due to model limitations. Secondary periods were randomly removed and the process was repeated 100 times for simulations 1 to 3. All analyses and modeling were conducted using R 3.1.2 software package (R Core Team 2014) unless otherwise noted.

### 2.5 Detecting trends in population abundance and estimating apparent survival rates

Each of the capture history datasets applied Robust Design capture–recapture models with the *RMark* interface (Laake, 2013). *RMark* provides an R‐based interface linked to the program Mark (White & Burnham, 1999). Based upon previous modeling work (Sprogis et al., 2016a), models were fit to constant survival (φ(.)), time‐varying Markovian emigration (γ″(*t*) ≠ γ′(*t*)), and time‐varying capture probabilities within primary periods (*p* = *c*(*t*,*s*)) (e.g., the best‐fitting model for adults and juveniles was φ(.) γ″(*t*) ≠ γ′(*t*) *p* = *c*(*t*,*s*)). The coefficient of variation (CV) and survival rate estimation for each model run were retained. The average CV for each scenario was calculated and used in analysis for detecting trends in population abundance.

Gerrodette's inequality model (Gerrodette, 1987) was applied following the method presented in Tyne et al. (2016). For each of the four scenarios, we used the average CV using the software package *Trends* (Gerrodette, 1993) to quantify the time required under each scenario to detect a 5% and 10% change in abundance at a statistical power of 0.8 and 0.95.

### 2.6 Quantifying effect of modified survey effort on the ability to conduct home range analyses

We used four metrics to explore the effect of survey effort on the ability to estimate individual dolphin home range size. Specifically, these metrics are based on previous recommendations by Seaman et al. (1999) who documented a minimum individual sighting threshold of ≥30, and ideally ≥50 sightings, for home range analyses of bottlenose dolphins to accurately represent of an individual's range. The first two metrics were therefore the number of individuals that were sighted on ≥30 occasions (following Sprogis et al., 2016b), and the number of individuals sighted on ≥50 occasions per simulation run over a 3‐year period. Further, we explored the effect of survey effort on the number of years for an individual to be documented on ≥30 and ≥50 occasions.

### 2.7 Quantifying effects of reduced survey effort on documenting calving events

We quantified the effect of reduced survey effort on our ability to detect calving events in the first year of life. We utilized sighting histories containing for reproductively active females during this study (December 2009 to November 2012). When a female was photographed with her dependent calf, the calf's presence was added to the sighting history. Following the same procedure used for the capture–recapture modeling, we then simulated reduced survey effort with one, two, or three secondary periods removed from each primary period (simulations 1–3 respectively). This process was repeated 100 times for each simulation of survey effort. From the Original Data set and for each simulation, the number of calves documented in the year in which they were born was retained.

## 3 RESULTS

### 3.1 Survey effort and summary statistics

From December 2009 to November 2012, we completed twelve primary periods each consisting of six secondary periods, resulting in a total of 72 secondary sampling periods. Completion of each secondary period was weather‐dependent and required 1–12 (4.07 ± 0.24 *SE*) days to complete. A total of 201 highly and moderately distinctively marked individual dolphins were documented. The average number of times each dolphin was sighted was 11.63 (±9.57 *SD*; range: 1–42). In each secondary period, the number of identified dolphins varied between 8 and 59 individuals (32.47 ± 1.61 *SE*).

### 3.2 Ability to detect changes in abundance based on survey effort

**Figure 2:**
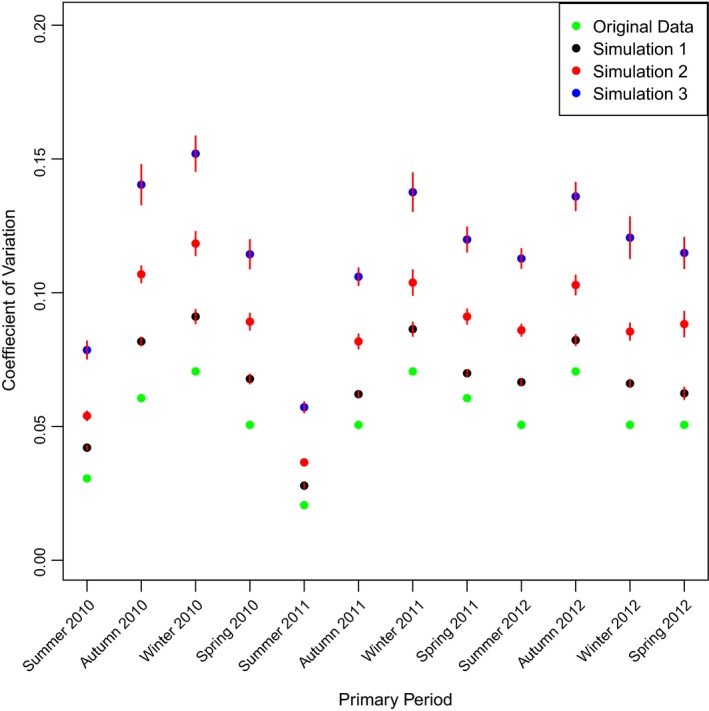
The average coefficient of variation (CV) for population abundance estimates for each primary period and each survey effort scenario from December 2009 (Summer 20/10) to November 2012 (Spring 2012). Reduced survey effort was simulated 100 times for Simulation 1 (five secondary periods per primary period) to Simulation 3 (three secondary periods per primary period). Error bars show the 95% confidence intervals

Under the Original Data, the precision of abundance estimates was high with an average CV (precision) of 0.05. Reduction of survey effort by one, two, or three secondary periods increased the average CV to 0.07, 0.08, and 0.12, respectively (Table 1; Figure 2). Detection of a 5% annual change in population abundance at 80% power would take 2.75–5.5 years (Original Data and Simulation 3, respectively) and 3.25–6.5 years at 95% power (Original Data and Simulation 3, respectively; Table 1). In 1.75 (Original Data) to 3.5 years (Simulation 3), we detected a 10% annual change in abundance at 80% power. The time to detect the same change at 95% power increased from 2 to 4.25 years (Table 1). Population abundance could have declined by 36% at the time of detection under reduced survey effort (Simulation 3; Table 1).

**Table 1:** Number of years to detect change in population abundance, percent decline/increase at the time of detection at two annual rates of change (0.05 and 0.1) at power = 95% or power = 80% with four (seasonal) abundance estimates per year

Scenario	Average CV	Power	Annual rate of change	Number of years needed to detect change	% decline at time of detection	% increase at time of detection
Original Data	0.05	0.8	0.05	2.75	−13	14
		0.95	0.05	3.25	−15	17
		0.8	0.1	1.75	−17	18
		0.95	0.1	2	−19	21
Simulation 1	0.07	0.8	0.05	3.5	−16	19
		0.95	0.05	4.25	−20	23
		0.8	0.1	2.25	−21	24
		0.95	0.1	2.75	−25	30
Simulation 2	0.08	0.8	0.05	4	−19	22
		0.95	0.05	5	−23	28
		0.8	0.1	2.75	−25	30
		0.95	0.1	3.25	−29	36
Simulation 3	0.12	0.8	0.05	5.5	−25	31
		0.95	0.05	6.5	−28	37
		0.8	0.1	3.5	−31	40
		0.95	0.1	4.25	−36	50

*Note*. Original Data = six original secondary periods, Simulation 1 = five randomly subsampled secondary periods, Simulation 2 = four randomly subsampled secondary periods, and Simulation 3 = three randomly subsampled secondary periods.

### 3.3 Estimation of apparent survival rates based on survey effort

**Figure 3:**
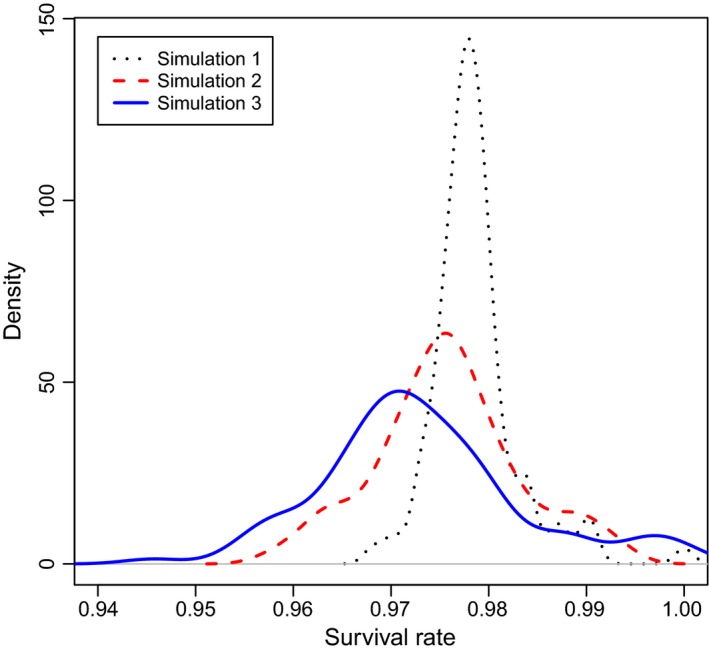
The density of occurrence of estimated annual apparent survival rates from 100 simulations for three scenarios of reduced survey effort. Surveys were structured following Pollock's Robust Design, with 12 primary periods between which the population was considered “open,” each consisting of six secondary periods (Original Data). Reduced survey effort was simulated by randomly removing one (Simulation 1), two (Simulation 2), or three (Simulation 3) secondary periods from each primary period

The annual apparent survival rate estimated based on the Original Data set was 0.981 (±0.005 *SE*; 95% CI 0.969–0.989). The range of apparent survival rate estimates was widest when survey effort was reduced by half (Simulation 3; range: 0.949–1) and narrowed as survey effort increased (Simulation 1; range: 0.971–0.995). The distributions of the estimated annual apparent survival rate overlapped substantially, indicating no strong difference in survival rate estimation between the four survey efforts examined (Figure 3).

### 3.4 Ability to conduct home range analyses based on survey effort

Thirteen individuals were seen on ≥30 occasions in the 3‐year study period of the Original Data set. Under all scenarios, no individual was observed on ≥50 occasions throughout the 3‐year period (Table 2). Reducing survey effort by one secondary period per primary period (Simulation 1) resulted in a decrease to 5.55 individuals (average) sighted ≥30 times (Table 2). When survey effort was reduced by half (Simulation 3), no individual was seen on ≥30 occasions (Table 2).

**Table 2:** The number of individual dolphins per simulation that were sighted on ≥30 and ≥50 occasions, respectively

Effort	Average number of individuals sighted on >30 occasions (±*SD*)	Average number of individuals sighted on >50 occasions
Original Data	13	0
Simulation 1	5.55 (±0.93)	0
Simulation 2	0.29 (±0.50)	0
Simulation 3	0	0

*Note*. Original Data are the original dataset (six secondary periods), while simulations 1–3 are results from 100 simulations and with survey effort reduced from five secondary periods to three secondary periods per primary period.

**Figure 4:**
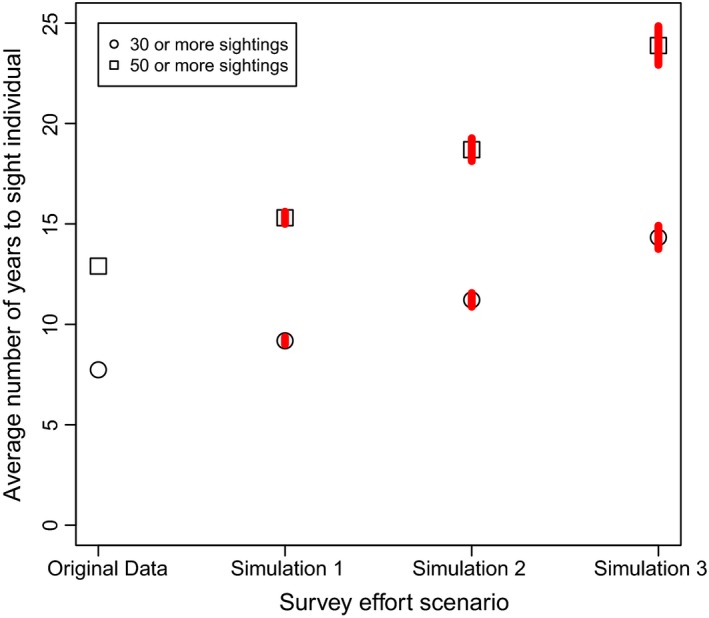
The average number of years required for an individual dolphin to be sighted on >30 and >50 occasions. Original Data are the results based on the original data set (six secondary periods per primary period), while simulations 1–3 are simulated to reduce survey effort from five to three secondary periods per primary period. Red error bars indicate the mean ± *SD*

Average sighting frequency during the 3‐year study period ranged from a minimum of 6.29 (±0.25 *SD*) sightings under Simulation 3 to a maximum of 11.63 sightings within the Original Data. Based on these results, it would take between 7.74 (Original Data) and 14.33 years (Simulation 3) for an individual, identified at the average sighting frequency, to be sighted ≥30 occasions (Figure 4). For an individual that was identified at the average sighting frequency, it would take 12.90 (Original Data) to 23.88 years (Simulation 3; Figure 4) to be seen ≥50 times.

### 3.5 Ability to detect calving events

**Figure 5:**
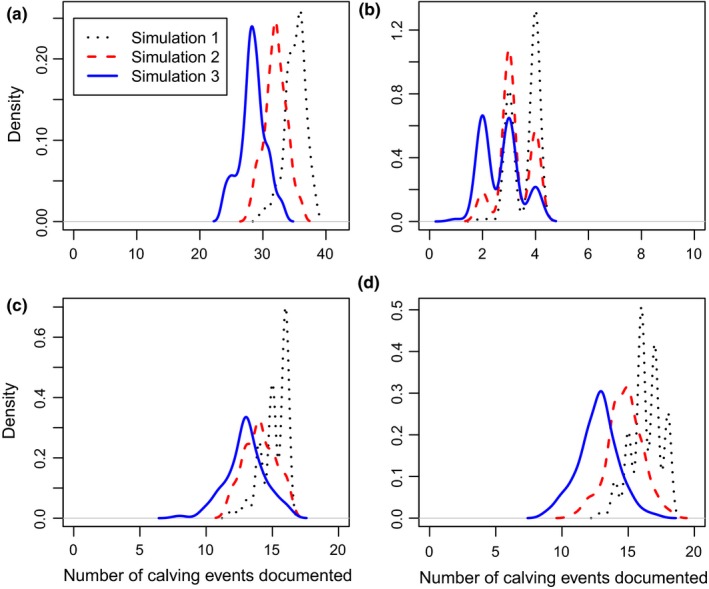
The density of occurrence of calving events documented in the year of birth across all simulations: Simulation 1 (five secondary periods per primary period); Simulation 2 (four secondary periods per primary period); and Simulation 3 (three secondary periods per primary period) during: (a) the full 3‐year study period; (b) Year 1 of the study; (c) Year 2 of the study; and (d) Year 3 of the study. A total of 40 calving events occurred in the Original Dataset (six secondary periods per primary period. Calves = Year 1: 4; Year 2: 17; Year 3: 19)

Within the Original Data, 40 calving events were documented, with 4, 17, and 19 new calves documented in each year of the study, respectively. Simulation 1 resulted in, on average, 10.0%, 10.8%, and 14.1% of calves not being documented in the year they were born (Table 3; Figure 5). In contrast, Simulation 3 resulted in, an average, 32.8%, 23.7%, and 33.1% of calves undocumented in the year they were born (Table 3; Figure 5).

**Table 3:** The average number of calving events and proportion of calving events undetected in the year of their birth for each scenario over the 3‐year study period

Scenario	Year of study	Average no. of calving events documented (±*SD*)	Range	Average proportion of calving events not documented (±*SD*)	Range
Original Data	Total	40	—	—	—
	Year 1	4	—	—	—
	Year 2	17	—	—	—
	Year 3	19	—	—	—
Simulation 1	Total	35.09 ± 1.65	30–38	12.28 ± 4.13%	5–25%
	Year 1	3.6 ± 0.51	2–4	10.00 ± 12.81%	0–50%
	Year 2	15.16 ± 0.98	12–16	10.82 ± 5.78%	5.88–29.41%
	Year 3	16.33 ± 1.16	13–18	14.05 ± 6.08%	5.26–31.58%
Simulation 2	Total	32.01 ± 1.76	28–36	19.98 ± 4.40%	10–30%
	Year 1	3.21 ± 0.61	2–4	19.75 ± 15.20%	0–50%
	Year 2	14.06 ± 1.19	12–16	17.29 ± 6.99%	5.88–29.41%
	Year 3	14.74 ± 1.32	11–18	22.42 ± 6.92%	5.26–42.11%
Simulation 3	Total	28.39 ± 2.06	24–33	29.03 ± 5.15%	17.5–40%
	Year 1	2.69 ± 0.72	1–4	32.75 ± 18.01%	0–75%
	Year 2	12.98 ± 1.47	8–16	23.65 ± 8.65%	5.88–52.94%
	Year 3	12.72 ± 1.48	9–17	33.05 ± 7.81%	10.53–52.63%

*Note*. Original Data contained the data set consisting of six secondary periods per primary period, while simulations 1, 2, and 3 were simulated reduced survey effort by one, two, and three secondary periods per primary period, respectively.

## 4 DISCUSSION

Our results highlight the need for careful consideration into survey intensity when collecting data to estimate demographic parameters of long‐lived species. Findings presented here suggest that population abundance could decline by 36% by the time of detection under reduced survey effort (Simulation 3). Reduced survey intensity limited our ability to estimate home range sizes, by increasing the time for individuals to be sighted on ≥30 occasions (an often‐used metric for home range analyses) from 7.74 to 14.33 years and resulted in a quarter to a third of calves born being undocumented in their year of birth. All four survey effort scenarios tested in this study are high in intensity compared to what is typically feasible in cetacean studies. Even so, results clearly demonstrate the importance of survey effort on the ability to assess demographic parameters with clear implications for population viability analyses, population forecasting, and conservation efforts to manage human–wildlife interactions.

### 4.1 Ability to detect changes in abundance under current and alternative survey effort

The ability to detect trends in abundance of marine mammals is limited in many cases. Except for pinnipeds, marine mammal monitoring programs have not been able to detect population declines in ≥50% of all taxonomic groups examined (Taylor, Martinez, Gerrodette, Barlow, & Hrovat, 2007). Compared to other studies (e.g., Brown et al., 2015; Parra et al., 2006; Tyne et al., 2016; see Table 4), the precision of our abundance estimates was high at current survey effort (Original Data). When survey effort was reduced by half (Simulation 3), 6.5 years were required to detect a 5% change in population abundance at 95% power. Previous studies found similar time periods required, with 7 years for Hawaiian spinner dolphins (*Stenella longirostris*; Tyne et al., 2016), 4 or 10 years for Indo‐Pacific bottlenose dolphins (Ansmann et al., 2013), 6 years for snubfin dolphins (*Orcaella heinsohni*; Parra et al., 2006), and 10 years for humpback dolphins (*Sousa sahulensis*; Parra et al., 2006; Table 4). Findings presented in this study suggest that reducing survey effort by half (Simulation 3) could result in a population abundance decline of 36% at the time of detection. The lack of ability to readily detect significant abundance decline significantly hinders the implementation of effective management efforts.

**Table 4:** Overview of delphinid studies that assessed the ability to detect changes in population abundance, with the coefficient of variation (CV) and the number of years to detect a change in abundance displayed

Species	Location	Study duration, number of abundance estimates, and sampling frequency	CV	Years to detect 5% change in abundance at 95% power (years)	Study
*Stenella longirostris*	Kona, Hawai`i	2 years, 2 estimates, 144 surveys per estimate	0.09	7	Tyne et al. (2016)
*Sousa sahulensis*	Cygnet Bay, Australia	2 years, 4 estimates, 5 surveys per estimate	0.117	9	Brown et al. (2015)
*Orcaella heinsohni*	Cygnet Bay, Australia	2 years, 4 estimates, 5 surveys per estimate	0.073	6	Brown et al. (2015)
*Orcaella heinsohni*	Roebuck Bay, Australia	2 years, 2 estimates, 7 surveys per estimate	0.124	9	Brown et al. (2015)
*Tursiops aduncus*	Cygnet Bay, Australia	2 years, 4 estimates, 5 surveys per estimate	0.14	11	Brown et al. (2015)
*Tursiops aduncus*	Beagle Bay, Australia	2 years, 2 estimates, 5 surveys per estimate	0.205	14	Brown et al. (2015)
*Sousa sahulensis*	Cleveland Bay, Australia	4 years, 4 estimates, ~110–210 survey hours per estimate	0.14	10	Parra et al. (2006)
*Orcaella heinsohni*	Cleveland Bay, Australia	4 years, 4 estimates, ~110–210 survey hours per estimate	0.08	6	Parra et al. (2006)
*Tursiops aduncus*	South Moreton Bay, Australia	2 years, 4 estimates, 15–26 surveys per estimate	0.03	4	Ansmann et al. (2013)
*Tursiops aduncus*	North Moreton Bay, Australia	2 years, 4 estimates, 15–26 surveys per estimate	0.12	10	Ansmann et al. (2013)
*Tursiops truncatus*	Moray Firth, Scotland	4 years, 4 estimates, 11–21 surveys per estimate	0.07	8	Wilson et al. (1999)

### 4.2 Effects of reduced survey effort on estimates of apparent survival

The apparent survival rates estimate here are similar to those documented for other free‐ranging dolphin populations (e.g., Brown et al., 2015; Ryan, Dove, Trujillo, & Doherty, 2011; Tezanos‐Pinto et al., 2013; Tyne, Pollock, Johnston, & Bejder, 2014), including those previously published for the Bunbury dolphin population (Smith et al., 2013; Sprogis et al., 2016a). Across all scenarios, apparent annual survival rates ranged from 0.949 to 1, with little difference as survey effort decreased. One possible explanation for this is that the original apparent survival rates were high due to the structure of the capture history and resighting rates of individuals (Original Data; 0.98); consequently, the removal of secondary periods resulted in estimates being relatively insensitive to the simulated reduction of survey effort. A reduction of survey effort was unlikely to alter the conclusions drawn pertaining to apparent annual survival rates for the population or to trigger alternative management approaches on its own. However, when combined with other demographic parameters (such as calf survival rates or fecundity estimates), a decrease in the estimated apparent survival rate may alter model forecasts in population viability analyses (Currey, Dawson, & Slooten, 2009; Manlik et al., 2016).

### 4.3 Implications for conducting home range analyses

Our results highlighted two potential limitations of reducing survey effort for conducting home range analyses. Previous research highlighted the importance of having ≥30 independent sightings of an individual (and preferable ≥50 sightings) to accurately estimate its home range size (Seaman et al., 1999). Our findings suggest that reducing survey effort from six secondary sampling periods per primary period (Original Data) to three secondary sampling periods (Simulation 3) increased the time required for an individual to be sighted on ≥30 occasions from 7.74 years (Original Data) to nearly 15 years (Simulation 3; Figure 4). Similarly, the time for an individual to be observed on ≥50 occasions increased from an average of 12.90 years (Original Data) to 23.88 years (Simulation 3; Figure 4). A natural consequence of the inability of a sampling design to exceed a minimum individual sighting frequency is the concurrent decline in total sample size for analyses (i.e., in the case the number of individuals for which home range size can be estimated). An increased number of years required to gather sufficient data to conduct home range analyses would eliminate our ability to examine whether individuals alter their usage of an area as the result of perturbation in their environment (Börger et al., 2006; Sprogis et al., 2016b).

### 4.4 The effect of survey effort on detecting calving events

Many resident cetacean populations exhibit seasonality in breeding and calving which typically peak during warmer months, with some out‐of‐season births occurring (Clapham, Young, & Brownell, 1999; Haase & Schneider, 2001; Hohn, Read, Fernandez, Vidal, & Findley, 1996; Mcguire & Aliaga‐rossel, 2007; Smith, Frère, Kobryn, & Bejder, 2016; Sørensen & Kinze, 1994; Steiner & Bossley, 2008). The weaning age of bottlenose dolphins (*Tursiops* sp.) is typically between ages of 3 and 6 years (Mann et al., 2000; Wells, 2014), with the majority weaned by 4 years of age (Mann et al., 2000). For some bottlenose dolphin populations, first‐year calf mortality ranges between 15% and 42% (Mann et al., 2000; Wells, 2014), with over 40% of calves not surviving to weaning (Mann et al., 2000). This highlights that a female dolphin could give birth to a calf and the calf could die before being documented via the research monitoring program. Our simulations quantified the effect of reduced survey effort on our ability to determine the timing of new calving events. Short‐time intervals between successive surveys will optimize documentation of calving events. Year‐round survey effort at reduced levels of survey effort was not enough to ensure that all calving events were documented in the first year of birth. Our results highlight that sustained high levels of year‐round survey effort were required, as reduced survey effort resulted in 5%–40% of calving events not being documented in the year during which they occurred.

Undocumented calving events will have multiple effects on estimates of demographic parameters. For example, intercalving intervals will be positively biased (Mann et al., 2000), resulting in lower fecundity or reproductive output estimates (Arso Civil, Cheney, Quick, Thompson, & Hammond, 2017). Missed calving events would also positively bias calf survival rates as the true calf mortality rate would be higher than that estimated (Mann et al., 2000). Consequently, utilizing parameters estimated from reduced survey effort are likely to result in inaccurate forecasts being made about the long‐term viability of a population (Currey et al., 2009; Manlik et al., 2016). The impacts of which can reduce the ability of a long‐term dataset to accurately inform management and policy decisions (Hughes et al., 2017).

### 4.5 Applicability of methods to other study systems

Long‐term monitoring programs serve a critical role in informing wildlife policymaking and our understanding of ecological systems (Editorial 2017; Hughes et al., 2017; Mann & Karniski, 2017). Such programs are costly (Tyne et al., 2016), and securing funding is an ongoing challenge (Editorial 2017; Hughes et al., 2017). Consequently, understanding the potential implications of reduced survey effort resulting from limited resources on the ability to estimate population demographic parameters is necessary. The methods presented here are broadly applicable to a wide range of study systems. The ability to detect changes in population abundance is applicable for any study system where the precision (CV) of abundance estimates can be quantified (Ham & Pearsons, 2000; Johnson, Camp, Brinck, & Banko, 2006; Taylor et al., 2007). Further, in study systems where individuals are uniquely identifiable, from either natural (e.g., cetaceans, elephants, sharks; Casale, Mazaris, Freggi, Vallini, & Argano, 2009; Holmberg, Norman, & Arzoumanian, 2009; Würsig & Jefferson, 1990) or artificial markings (e.g., birds, seals, turtles; Casale et al., 2009; McMahon, van den Hoff, & Burton, 2005; Ruiz‐Gutierrez et al., 2012), the subsampling procedure presented here can be used to assess the implications of reduced survey effort for conducting home range analyses. Lastly, in study systems with uniquely identifiable individuals where parental care is provided to young (e.g., birds and mammals), the potential consequences of reduced survey effort on the ability to detect reproductive events can be quantified using the methods applied in this study.

## 5 CONCLUSIONS

Our results highlight the effect of survey intensity on estimating abundance and demographic parameters of a long‐lived marine top predator. All four survey effort scenarios tested in this study are relatively high in sampling frequency (12–24 surveys of the entire study area per year) compared to what is typically logistically and financially feasible in cetacean studies. Even so, our results demonstrate the consequences of reducing survey intensity on the ability to (a) detect trends in population abundance; (b) conduct home range analyses; and (c) accurately estimate reproductive outputs. The ability to reliably assess potential population consequences of human activities on wildlife populations is dependent on accurate measures of their vital rates. Incorrect estimates of trends in abundance, survival, and reproduction parameters will affect population viability forecasts. Inaccurate population viability assessments may lead to declining populations being misidentified as stable or increasing, or vice versa, resulting in erroneous advice for management and policy implementation. Our results present the importance of considering the level of required effort during survey design, and implications of survey effort on informing population viability analyses, population forecasting, and conservation efforts to manage wildlife of commercial, natural, and cultural significance.

## AUTHOR'S CONTRIBUTIONS

LB and JS conceived and designed the experiments. KS collected and processed the data. JS analyzed the data. JS and LB wrote the manuscript, with contributions to drafting, review, and editorial input from KS.

## DATA ACCESSIBILITY

Data available from the Dryad Digital Repository: https://doi.org/10.5061/dryad.6381477.
